# Gallbladder Stone Disease Is Associated with an Increased Risk of Migraines

**DOI:** 10.3390/jcm7110455

**Published:** 2018-11-21

**Authors:** Chien-Hua Chen, Cheng-Li Lin, Chia-Hung Kao

**Affiliations:** 1 Digestive Disease Center, Changbing Show-Chwan Memorial Hospital, Lukang 505, Taiwan; showchench@yahoo.com.tw; 2 Digestive Disease Center, Show-Chwan Memorial Hospital, Changhua 500, Taiwan; 3 Department of Food Science and Technology, Hungkuang University, Taichung 404, Taiwan; 4 Management Office for Health Data, China Medical University Hospital, Taichung 404, Taiwan; orangechengli@gmail.com; 5 College of Medicine, China Medical University, Taichung 404, Taiwan; 6 Graduate Institute of Biomedical Sciences and School of Medicine, College of Medicine, China Medical University, Taichung 404, Taiwan; 7 Department of Nuclear Medicine and PET Center, China Medical University Hospital, Taichung 404, Taiwan; 8 Department of Bioinformatics and Medical Engineering, Asia University, Taichung 404, Taiwan

**Keywords:** gallbladder stone disease, migraine, comorbidity, cohort study, cholecystectomy

## Abstract

**Background:** Several pathophysiological mechanisms are shared in both gallbladder stone disease (GSD) and migraines. We assessed the migraine risk for patients diagnosed with GSD. **Methods:** We identified 20,427 patients who were diagnosed with GSD between 2000 and 2011 from Taiwan’s National Health Insurance Research Database (NHIRD) as the study cohort. We randomly selected 81,706 controls from the non-GSD population with frequency matching by age and index year for the control cohort. All patient cases were followed until the end of 2011 to measure the incidence of migraines. **Results:** The cumulative incidence of migraines was greater in patients with GSD than in those without GSD (log-rank test: *p* < 0.001). The risk of migraine (3.89 vs. 2.30 per 10,000 person-years, adjusted hazard ratio (aHR) = 1.56, 95% confidence interval (CI) = 1.41–1.73) was greater for the GSD cohort than that for the non-GSD cohort. The risk of migraine increased with the time of follow-up after a diagnosis of GSD. The risk of migraine contributed by GSD was greater for all age groups. The risk of migraine for GSD patients with depression (aHR = 2.89, 95% CI = 2.21–3.77), anxiety (aHR = 2.07, 95% CI = 1.58–2.70), and coronary artery disease (CAD) (aHR = 2.05, 95% CI = 1.69–2.48) tended to be greater than that for GSD patients without depression (aHR = 1.54, 95% CI = 1.39–1.72), anxiety (aHR = 1.62, 95% CI = 1.46–1.81), and CAD (aHR = 1.65, 95% CI = 1.47–1.85), respectively. Compared with the patients without GSD, the risk of developing migraines was greater in those GSD patients either with (aHR = 1.39, 95% CI = 1.19–1.63) or without (aHR = 1.67, 95% CI = 1.48–1.88) cholecystectomy. Compared with the GSD patients that have not had a cholecystectomy, the risk of developing migraines was lower in the GSD patients that had a cholecystectomy (aHR = 0.83, 95% CI = 0.69–0.99). **Conclusions:** GSD is associated with an increased risk of migraines in the Taiwanese population, but the risk diminishes after a cholecystectomy. Furthermore, in the development of migraines, GSD is synergic with some migraine-associated comorbidities, such as CAD, depression, and anxiety. Further study is necessary to clarify whether GSD is a causal risk factor for migraine.

## 1. Introduction

A migraine is characterized by repeated attacks of headaches, lasting 4–72 h, in patients who appear normal under physical examination and who exhibit no other obvious cause for the headache. Moreover, a migraine has at least two of unilateral pain, throbbing pain, and aggravation by movement, and is characterized by at least one of nausea or vomiting, and photophobia or phonophobia [1]. A migraine, along with quadriplegia, psychosis, and dementia, has been deemed one of the most disabling chronic disorders [2]. The reported prevalence of migraines in adults ranges from 3.3% to 21.9% for women and from 0.7% to 16.1% for men [3]. Migraines are a major cause of decreased productivity and reduced health-related quality of life; its impacts on patients are sufficiently severe and widespread to impose notable socioeconomic burdens on communities. To facilitate the clinical implications of the preventive programs and treatment strategies for migraines, it is important to find more migraine-related factors although migraines have been suggested to be related to female sex, obesity, diabetes, cardiovascular disorder, and psychosocial stress [4].

Gallbladder stone disease (GSD) is a common gastrointestinal disorder worldwide, and its prevalence increases with the degree of socioeconomic development. It is present in 10% of the populations of Western countries and 3–10% of the populations of Asian countries; 5% of adults in Taiwan suffer from GSD [5]. In an observational study of the natural history of GSD, 2% of the asymptomatic GSD patients developed a new-onset biliary colic per year for the first 5 years, with a total incidence of 15% at 10 years of follow-up [6]. However, GSD is a major public health concern because it sometimes causes complications, such as cholecystitis, pancreatitis, cholangitis, gallstone ileus, and gallbladder empyema or perforation. Similar to migraines, GSD has been suggested to be related to female sex, obesity, diabetes, hyperlipidemia, and cardiovascular disorder [7]. The prevalence of GSD is expected to increase with the increasing prevalence of metabolic syndrome worldwide [8]. 

Migraines and GSD share many risk factors, particularly the metabolic disorders, and the association between migraines and upper abdominal symptoms implies a common pathophysiological mechanism between migraines and biliary tract disorders [9]. Furthermore, cholecystokinin (CCK) and calcitonin gene-related peptide (CGRP) may lead to the pathophysiological mechanisms shared by migraines and GSD [9,10,11]. A decreased plasma CCK A receptor of the gallbladder has been observed in GSD patients, resulting in impaired postprandial gallbladder emptying and increased plasma CCK levels [11]. However, a dilating effect on arterial vessels after postprandial-increased CCK concentration was recognized and proposed as a possible pathogenesis for migraine attacks [12]. CGRP can antagonize CCK to diminish the smooth muscle tone of the gallbladder and the plasma level of CGRP is increased in GSD patients [11]. Furthermore, CGRP can coexist with CCK in the trigeminal perivascular fibers to trigger migraine attacks by increasing cerebral blood flow [13]. The bidirectional interaction between GSD and psychosocial stress has been well-recognized since GSD patients tend to be obsessive and subordinate, and an increased sympathetic tone can disturb gallbladder motility [14,15]. Reciprocal interaction between migraine and anxiety or depression has also been recognized as the main pathogenesis for the relation of somatic headache to the dysfunctional serotoninergic and dopaminergic systems [16,17]. Moreover, anxiety can activate the vasodilatory effect of CCK to facilitate pain transmission [18]. However, no literature discusses the relationship between GSD and the development of migraines despite several pathophysiological mechanisms shared in both GSD and migraine. 

In this study, we hypothesized that a history of GSD might be associated with an increased risk of migraine development. We conducted a nationwide population-based cohort study by analyzing data from the National Health Insurance Research Database (NHIRD) of Taiwan to determine the relationship of GSD to the subsequent development of migraines and to assess the risk difference of migraines between GSD patients with and without having had a cholecystectomy.

## 2. Methods

### 2.1. Data Source

Since 1995, all residents of Taiwan have been required by law to be enrolled in the National Health Insurance (NHI) program. In 2014, the NHI program coverage rate was 99.9% [19]. The NHIRD of Taiwan is a nationwide claims database, maintained by the National Health Research Institutes. We used the Longitudinal Health Insurance Database 2000 (LHID2000), which comprises one million insurants in the NHIRD; the LHID2000 includes the medical records of all beneficiaries from the beginning of 1996 to the end of 2011. We randomly selected from the year 2000 Registry for Beneficiaries; all data were deidentified to ensure patient anonymity. This retrospective cohort study complied with the guidelines of the Declaration of Helsinki and was approved by the Research Ethics Committee of China Medical University (CMUH104-REC2-115-CR3). 

### 2.2. Sample Participants

Patients newly diagnosed with gallbladder stone disease (GSD) (ICD-9 574.0, 574.1, 574.2, 574.6, 574.7, 574.8, and 574.9) from 2000 to 2011 were identified from LHID2000. The index date was set as the date of GSD diagnosis. Moreover, we divided the patients with gallstones into two groups according to the history of having had a cholecystectomy or not. Patients with a history of migraines (ICD-9-CM code 346) diagnosed before the index date, aged younger than 20 years, or missing information for age or sex were excluded. For each GSD case, four non-GSD controls without a history of cholelithiasis (ICD-9-CM code 574) or migraines were identified from LHID2000, and were frequency-matched to the case with regard to age (every 5-year span), sex, and year of GSD diagnosis. 

### 2.3. Outcome and Comorbidities

All participants were followed from the index date until the occurrence of migraine, withdrawal from the NHI, or the end of 2011, whichever came first. The pre-existing comorbidities included diabetes (ICD-9-CM code 250), hypertension (ICD-9-CM codes 401–405), hyperlipidemia (ICD-9-CM code 272), stroke (ICD-9-CM codes 430–438), chronic obstructive pulmonary diseases (COPD) (ICD-9-CM codes 491, 492, 496), coronary artery disease (CAD) (ICD-9-CM codes 410–414), depression (ICD-9-CM codes 296.2, 296.3, 300.4, 311), and anxiety (ICD-9-CM code 300.00).

### 2.4. Statistical Analysis

Distributions of demographic characteristics, including age, sex, and comorbidities, were compared between GSD patients and the non-GSD cohort by using chi-squared tests. Incidence densities of migraines for the two cohorts were calculated by age, sex, and comorbidity. To estimate the cumulative incidence rates of migraines in the GSD cohort and the non-GSD cohort, we performed a survival analysis using the Kaplan–Meier method, with significance based on the log-rank test. Both univariable and multivariable versions of Cox’s proportion hazard regression were used to examine the effect of GSD on the risk of migraine, as shown by hazard ratio (HR) with 95% confidence intervals (CI). The risk difference of migraine between GSD patients with and without having had a cholecystectomy was also assessed in our study. All analyses were performed with SAS statistical software (Version 9.4 for Windows; SAS Institute, Inc., Cary, NC, USA). A two-tailed *p* < 0.05 was considered statistically significant. 

## 3. Results

**Figure 1:**
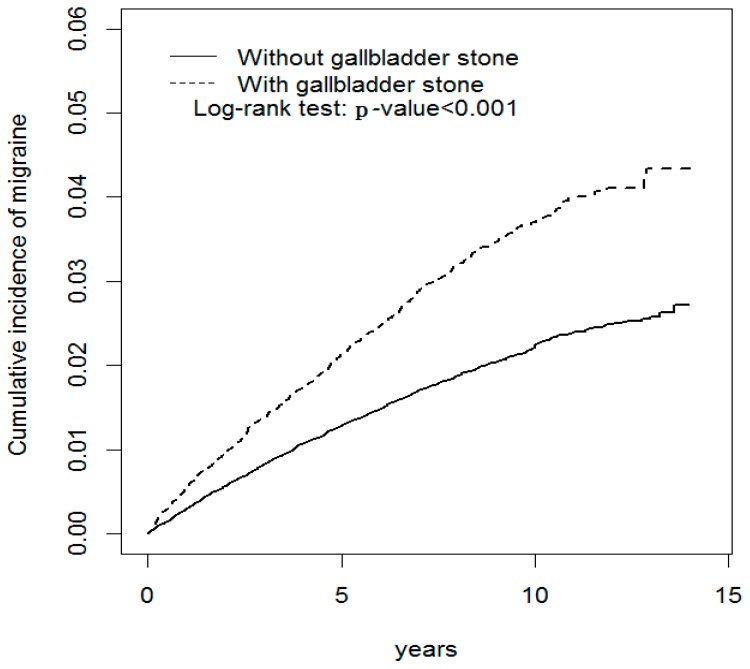
Cumulative incidence comparison of migraines for patients with (dashed line) or without (solid line) gallbladder stone disease.

Demographic characteristics and comorbidity values for both study cohorts are presented in Table 1. The mean age of the non-GSD cohort was 55.2 ± 16.0 years and that of the GSD cohort was 55.9 ± 15.7 years, with 37.7% of them aged ≤49 years. Females accounted for 54.2% of the study patients. Compared with the non-GSD cohort, the GSD cohort had a higher prevalence of diabetes, hypertension, hyperlipidemia, stroke, COPD, CAD, depression, and anxiety (all *p* < 0.001). The mean follow-up time was 6.74 (SD = 3.89) years in the GSD cohort and 6.83 (SD = 3.91) years in the non-GSD cohort. The log-rank test results for the cumulative incidence of migraines are shown in Figure 1. The GSD patients had a significantly higher migraine incidence than that of the patients in the non-GSD cohort (log-rank *p* < 0.001).

The overall incidence density rate of migraine in the GSD cohort was 3.89 per 10,000 person-years and 1.69-fold higher than that in the non-GSD cohort (2.30 per 10,000 person-years), with an adjusted HR (aHR) of 1.56 (95% CI = 1.41–1.73) (Table 2). In a stratified analysis for age, the risk of migraine in the GSD cohort relative to that in the non-GSD cohort was significantly greater for all age groups. In a stratified analysis for sex, the aHR was significantly higher both in women (aHR = 1.49, 95% CI = 1.32–1.68) and in men (aHR = 1.77, 95% CI = 1.46–2.15). We stratified patients by comorbidity: the risk of migraine was 1.87-fold higher in the GSD cohort without comorbidity than in the non-GSD cohort without comorbidity (95% CI = 1.60–2.19). Likewise, the risk of developing migraines was especially higher in GSD patients with comorbidity (aHR = 1.38, 95% CI = 1.21–1.58). However, it should be noted that the relative migraine risk contributed by GSD was greater for patients without comorbidity.

In the multivariable model, GSD was associated with the development of migraines (aHR = 1.56, 95% CI = 1.41–1.73) (Table 3). The risk of developing migraines decreased with each one-year increment of age (aHR = 0.99, 95% CI = 0.98–0.99) and was 2.16-fold higher for women than for men (95% CI = 1.95–2.4). The risk of developing migraines was greater for patients with comorbidity of CAD (aHR = 1.44, 95% CI = 1.26–1.64), depression (aHR = 1.75, 95% CI = 1.45–2.11), and anxiety (aHR = 1.60, 95% CI = 1.35–1.89).

Table 4 presents the joint effects of GSD and CAD, depression, and anxiety on migraine risk. We observed that the migraine risk for GSD patients with depression (aHR = 2.89, 95% CI = 2.21–3.77), anxiety (aHR = 2.07, 95% CI = 1.58–2.70), and CAD (aHR = 2.05, 95% CI = 1.69–2.48) tended to be greater than that for GSD patients without depression (aHR = 1.54, 95% CI = 1.39–1.72), anxiety (aHR = 1.62, 95% CI = 1.46–1.81), and CAD (aHR = 1.65, 95% CI = 1.47–1.85), respectively.

Table 5 presents the incidence and hazard ratio of migraines associated with GSD patients with or without having had a cholecystectomy. Compared with the patients without GSD, the risk of developing migraines was greater in those GSD patients either with (aHR = 1.39, 95% CI = 1.19–1.63) or without (aHR = 1.67, 95% CI = 1.48–1.88) having had a cholecystectomy. However, compared with the GSD patients without having had a cholecystectomy, the risk of developing migraines was lower in the GSD patients that underwent a cholecystectomy (aHR = 0.83, 95% CI = 0.69–0.99).

## 4. Discussion

Our results support other findings stating that the prevalence of GSD increases with age and most (54.2%) of the GSD patients in our study were female. The aging process can increase biliary cholesterol secretion, decrease bile salts synthesis and secretion, impair gallbladder motility, and increase exposure time to the environmental factors to increase the relative risk of GSD [20]. Nonetheless, estrogen can upregulate the low-density lipoprotein cholesterol (LDL-C) receptor to increase free liver cholesterol secretion [7]. Compared with the non-GSD patients, our results suggest that the GSD patients had a higher number of comorbidities such as diabetes, hypertension, hyperlipidemia, stroke, COPD, CAD, depression, and anxiety. Diabetes with hyperinsulinemia, resulting from insulin resistance, tends to increase biliary secretion of cholesterol, inhibit bile acid secretion, and impair gallbladder motility [8,21]. Hypertension is supposed to cause GSD through increased sympathetic nervous activities [22]. Hypertriglyceridemia, rather than hypercholesterolemia, can increase the formation of GSD by causing bile supersaturation and impairing gallbladder contraction [23]. GSD and cardiovascular disorder (CVD) share several pathophysiological features, such as the precipitation of cholesterol in bile for GSD and the accumulation of cholesterol in atherosclerotic plaque for CVD, a low level of plasma insulin-like growth factor (IGF-1) leading to impaired gallbladder emptying for GSD and exacerbated atherosclerosis for CVD, and a high level of oxidative stress in the gallbladder mucosa for GSD and increased endothelial inflammation with high plasma homocysteine level for CVD [24,25,26,27,28,29,30]. COPD is recognized as a disease with proinflammatory and prothrombotic status, which tends to increase the development of GSD [31]. Finally, psychological stress can impair gallbladder emptying by increasing the sympathetic tone [14,15].

The risk of migraine was associated with GSD, female sex, CAD, depression, and anxiety in our study. The female predominance of migraine was suggested to be related to estrogen, which can increase nitric oxide synthesis in endothelial cells to increase vascular dilatation capacity [32,33]. Furthermore, high levels of estrogen can upregulate gene expression and intracellular signal-regulated kinase to intensify inflammation and neuropathic pain [34,35]. With more co-existing metabolic disorders and endothelial dysfunction, migraine has been reported to have a synergic effect with prothrombotic factors to increase the events of CVD [36]. With dopaminergic symptoms for the migraine prodrome and the serotoninergic effect of vasodilatation for the symptoms of headache, serotonin and dopamine are the main neurotransmitters involved in mood-triggered symptoms of migraine [13,37]. 

After adjustments for age, sex, and comorbidities of hypertension, hyperlipidemia, coronary artery disease, depression, and anxiety, our results reveal that GSD is related to the subsequent development of migraines. Moreover, our results also suggest that the risk of migraine increases with an increase in the time without a follow-up, after a diagnosis of GSD (Figure 1). The causal relationship between GSD and migraines could not be ascertained in our study, but our results support an increased risk of migraine after GSD diagnosis. In addition, our results demonstrate that GSD is synergic to migraine-associated comorbidities, such as CAD, depression, and anxiety, in contributing to the subsequent development of migraines (Table 3). We compared the incidence of migraines not only between patients with and without gallstones, but also between patients with and without having had a cholecystectomy to lower the risk of misclassification or surveillance bias for GSD (Table 5). It was noted that our findings consistently support the migraine risk was greater in those GSD patients either with or without having had a cholecystectomy than that in the patients without GSD, and these findings validate the close association between GSD and migraines. However, further study is necessary to determine whether GSD is a risk factor or an epiphenomenon for migraine development. 

The exact mechanisms for the association between GSD, cholecystectomy, and migraines remain undetermined. The possible explanations for the association between GSD and migraines may be shared risk factors between GSD and migraines, the common peptides such as CCK and CGRP involved in both the pathogenesis of GSD and that of migraines, and multidirectional interaction between GSD and migraine through psychosocial stress [9,10,11,12,13,14,15,16,17,18]. It is reasonable to assume that an increased migraine risk in GSD patients might be directly related to the effect of GSD since we have attempted to control the possible confounding effects of age, sex, and comorbidities of hypertension, hyperlipidemia, coronary artery disease, depression, and anxiety. Furthermore, our results indicate that the relative migraine risk contributed by GSD was significantly higher in patients without comorbidity (Table 2). Moreover, our results disclose that the migraine risk in patients with GSD diminished after having had a cholecystectomy after adjustment for age, sex, and comorbidities of hypertension, hyperlipidemia, coronary artery disease, depression, and anxiety. This finding supports a protective effect of cholecystectomy against the development of migraines in patients with GSD and portends that GSD per se may be an independent risk factor for the development of migraines, although the mechanism and the causal relationship could not be ascertained in this observational study. A cholecystectomy is mainly indicated for biliary complications such as cholecystitis, pancreatitis, or cholangitis in clinical settings [38]. In this way, GSD may be beyond a biliary tract disease and is a marker of migraine occurrence. However, cholecystectomies deserve more studies to change its clinical indication since having had a cholecystectomy will ameliorate the migraine risk, a disease with heavy socioeconomic burdens on communities. 

Our study has several strengths. First, our study is the first population-based study to examine the association between GSD and the subsequent development of migraines. Second, large data from a longitudinal database with a 12-year observation period for a representative cohort of 1,000,000 citizens strengthened the statistical power of the calculations. Third, the recruited patients were sampled from a stable population, namely, the approximately 99% of Taiwan residents who are covered by the Taiwan NHI program. Therefore, our findings can provide the generalizability to Taiwan. Finally, our longitudinal cohort study enabled assessing the temporal association between GSD and migraines; the association between GSD and migraines is supported in the present study. 

Our study has several limitations. First, we might have neglected potential confounding factors because the NHIRD cannot provide detailed information on migraine-related lifestyle, socioeconomic status, family history, and body mass index (BMI). However, we used the diagnosis of COPD to replace smoking habits for adjustment in this study, because COPD is well known to be related to smoking. In addition, BMI is closely related to diabetes, hypertension, hyperlipidemia, stroke, and CAD. The aforementioned factors were adjusted for the partial exclusion of the confounding effect of BMI. However, GSD was consistently related to migraine development in the multivariable Cox’s proportion hazard regression. Second, patients who did not seek medical consultation for GSD could not be recorded in our study. Because more than 99% of Taiwan’s population was covered by the NIH program and the well-established social benefit system, the accessibility and affordability of healthcare were both too high to compromise our findings. Moreover, we also compared the incidence of migraines between the clinically relevant gallstones patients with having had a cholecystectomy and without having had a cholecystectomy, rather than between the asymptomatic gallstones patients and general population, to lower the risk of surveillance bias, and our findings supported the ameliorating effect of having had a cholecystectomy against the development of migraines in patients with gallstones. However, migraines have been regarded as an under-identified and undertreated disease; therefore, the correlation between GSD and migraines might be underestimated in our study [39]. Finally, our observational study cannot provide the causal pathogenesis for the multidirectional relationship between GSD, cholecystectomy, and migraines.

## 5. Conclusions

In conclusion, the results from our population-based cohort study indicate that GSD is associated with an increased risk of developing migraine in the Taiwanese population and the risk diminished after having had a cholecystectomy. Furthermore, GSD was synergic to some migraine-associated comorbidities, such as CAD, depression, and anxiety, in the subsequent development of migraines. However, further study is necessary to clarify whether GSD is an epiphenomenon or independent risk factor for migraines.

## Figures and Tables

**Table 1:** Demographic characteristics and comorbidities in cohorts with and without gallbladder stone disease.

Variable	Gallbladder Stone Disease		*p*-Value
	No	Yes	
	*N* = 81,706	*N* = 20,427	
Age, year			0.99
≤34	8576 (10.5)	2144 (10.5)	
35–49	22,188 (27.2)	5547 (27.2)	
50–64	25,436 (31.1)	6359 (31.1)	
65+	25,506 (31.2)	6377 (31.2)	
Mean ± SD ^†^	55.2 (16.0)	55.9 (15.7)	<0.001
Sex			0.99
Female	44,248 (54.2)	11,062 (54.2)	
Male	37,458 (45.8)	9365 (45.8)	
Comorbidity			
Diabetes	6854 (8.39)	2642 (12.9)	<0.001
Hypertension	26,745 (32.7)	8448 (41.4)	<0.001
Hyperlipidemia	14,765 (18.1)	5622 (27.5)	<0.001
Stroke	3245 (3.97)	995 (4.87)	<0.001
Chronic obstructive pulmonary diseases	8211 (10.1)	2909 (14.2)	<0.001
Coronary artery disease	12,495 (15.3)	4862 (23.8)	<0.001
Depression	2803 (3.43)	1227 (6.01)	<0.001
Anxiety	4165 (5.10)	1783 (8.73)	<0.001

Chi-squared test; ^†^ *t*-test.

**Table 2:** Incidence of migraine by age, sex, and comorbidity, with Cox model-measured hazards ratios for patients with gallbladder stone disease compared with those without gallbladder stone disease.

	Gallbladder Stone Disease						Gallbladder Stone Disease to Non-Gallbladder Stone Disease	
	No			Yes				
Variables	Event	Person-Years	Rate ^#^	Event	Person-Years	Rate ^#^	Crude HR ^※^ (95% CI)	Adjusted HR ^†^ (95% CI)
All	1286	558,325	2.30	536	137,776	3.89	1.69 (1.52, 1.86) ***	1.56 (1.41, 1.73) ***
Stratify age								
≤34	115	57,278	2.01	50	14,645	3.41	1.70 (1.22, 2.37) **	1.59 (1.14, 2.24) **
35–49	414	165,593	2.05	190	40,652	4.67	1.86 (1.57, 2.21) ***	1.66 (1.39, 1.98) ***
50–64	455	183,146	2.48	184	44,704	4.12	1.65 (1.39, 1.96) ***	1.50 (1.26, 1.78) ***
65+	302	152,307	1.98	112	37,775	2.96	1.49 (1.20, 1.85) ***	1.41 (1.13, 1.75) **
Sex								
Female	961	311,909	3.08	382	76,969	4.96	1.61 (1.43, 1.81) ***	1.49 (1.32, 1.68) ***
Male	325	246,416	1.32	154	60,806	2.53	1.92 (1.58, 2.32) ***	1.77 (1.46, 2.15) ***
Comorbidity ^‡^								
No	614	324,976	1.89	209	58,059	3.60	1.90 (1.63, 2.23) ***	1.87 (1.60, 2.19) ***
Yes	672	233,349	2.88	327	79,716	4.10	1.43 (1.26, 1.64) ***	1.38 (1.21, 1.58) ***

Rate ^#^, incidence rate, per 10,000 person-years; Crude HR ^※^, relative hazard ratio; Adjusted HR ^†^: multivariable analysis including age, sex, and comorbidities of hypertension, hyperlipidemia, coronary artery disease, depression, and anxiety. ^‡^ Patients with any one of the comorbidities (diabetes, hypertension, hyperlipidemia, stroke, chronic obstructive pulmonary diseases, coronary artery disease, depression, and anxiety) were classified into the comorbidity group. ** *p* < 0.01, *** *p* < 0.001.

**Table 3:** Hazard ratios of migraine in association with sex, age, and comorbidities in univariable and multivariable Cox regression models.

	Crude HR ^※^		Adjusted HR ^†^	
Variable	HR	(95% CI)	HR	(95% CI)
Gallbladder stone disease	1.69	(1.52, 1.86) ***	1.56	(1.41, 1.73) ***
Age, years	0.99	(0.992, 0.998) **	0.99	(0.98, 0.99) ***
Sex (Female vs. male)	2.24	(2.02, 2.49) ***	2.16	(1.95, 2.40) ***
Baseline comorbidities (yes vs. no)				
Diabetes	0.84	(0.70, 1.02)	-	-
Hypertension	1.15	(1.05, 1.27) **	1.10	(0.97, 1.24)
Hyperlipidemia	1.18	(1.06, 1.32) **	0.98	(0.87, 1.11)
Stroke	0.79	(0.59, 1.07)	-	-
Chronic obstructive pulmonary diseases	1.12	(0.96, 1.30)	-	-
Coronary artery disease	1.49	(1.33, 1.67) ***	1.44	(1.26, 1.64) ***
Depression	2.41	(2.01, 2.87) ***	1.75	(1.45, 2.11) ***
Anxiety	2.25	(1.92, 2.63) ***	1.60	(1.35, 1.89) ***

Crude HR ^※^, relative hazard ratio; Adjusted HR ^†^: multivariable analysis including age, sex, and comorbidities of hypertension, hyperlipidemia, coronary artery disease, depression, and anxiety. ** *p* < 0.01, *** *p* < 0.001.

**Table 4:** Cox proportional hazard regression analysis for the risk of migraine-associated gallbladder stone disease with joint effects of comorbidity.

Variables		*N*	Event	Adjusted HR ^†^ (95% CI)
Gallbladder stone disease	Coronary artery disease			
No	No	69,211	1037	1 (Reference)
No	Yes	12,495	249	1.56 (1.33, 1.82) ***
Yes	No	15,565	394	1.65 (1.47, 1.85) ***
Yes	Yes	4862	142	2.05 (1.69, 2.48) ***
Gallbladder stone disease	Depression			
No	No	78,903	1215	1 (Reference)
No	Yes	2803	71	1.67 (1.30, 2.13) ***
Yes	No	19,200	475	1.54 (1.39, 1.72) ***
Yes	Yes	1227	61	2.89 (2.21, 3.77) ***
Gallbladder stone disease	Anxiety			
No	No	77,541	1176	1 (Reference)
No	Yes	4165	110	1.84 (1.50, 2.26) ***
Yes	No	18,644	475	1.62 (1.46, 1.81) ***
Yes	Yes	1783	61	2.07 (1.58, 2.70) ***

Adjusted HR ^†^: adjusted for age, sex and other comorbidities. *** *p* < 0.001.

**Table 5:** Incidence and hazard ratio of migraine associated with gallbladder stone disease patients with or without having had a cholecystectomy.

Variables	*N*	Event	PY	Rate ^#^	Adjusted HR ^†^ (95% CI)	Adjusted HR ^†^ (95% CI)
Gallbladder stone disease (No)	81,706	1286	558,325	2.30	1 (Reference)	
Gallbladder stone disease (Yes) Cholecystectomy (No)	12,675	354	84,399	4.19	1.67 (1.48, 1.88) ***	1 (Reference)
Cholecystectomy (Yes)	7752	182	53,377	3.41	1.39 (1.19, 1.63) ***	0.83 (0.69, 0.99) **

Rate ^#^, incidence rate, per 10,000 person-years; Adjusted HR ^†^: Multivariable analysis including age, sex, and comorbidities of hypertension, hyperlipidemia, coronary artery disease, depression, and anxiety. ** *p* < 0.01, *** *p* < 0.001.

## Abbreviations

GSD	Gallbladder Stone Disease
NHIRD	National Health Insurance Research Database
CAD	Coronary Artery Disease
aHR	Adjusted Hazard Ratio
CI	Confidence Interval
CCK	Cholecystokinin
CGRP	Calcitonin Gene Related Peptide
NHI	National Health Insurance
LHID2000	Longitudinal Health Insurance Database
ICD-9-CM	International Codes of Disease Ninth Edition Clinical Modification
COPD	Chronic Obstructive Pulmonary Disease
CAD	Coronary Artery Disease
LDL-C	Low-Density Lipoprotein Cholesterol
CVD	Cardiovascular Disorder
IGF-1	Insulin-Like Growth Factor-1
HDL-C	Low-Density Lipoprotein Cholesterol
BMI	Body Mass Index
